# Reliable relay assisted communications for IoT based fall detection

**DOI:** 10.1038/s41598-024-56124-z

**Published:** 2024-03-15

**Authors:** Khulud K. Alharbi, Sajid H. Alvi, Bakhtiar Ali, Jawad Mirza, Muhammad Awais Javed, Hatem A. Alharbi

**Affiliations:** 1 Department of Health Administration and Hospitals, College of Public Health and Health Informatics, Umm Al-Qura University, Makkah, Saudi Arabia; 2 Department of Physics, COMSATS University Islamabad, Islamabad, 45550 Pakistan; 3 Department of Electrical and Computer Engineering, COMSATS University Islamabad, Islamabad, 45550 Pakistan; 4 Department of Computer Engineering, College of Computer Science and Engineering, Taibah University, 42353 Madinah, Saudi Arabia

**Keywords:** IoT, fall detection, Non-orthogonal multiple access (NOMA), Amplify-and-forward (AF), Decode-and-forward (DF), Signal-to-interference-plus-noise ratio (SINR) analysis, Computer science, Electrical and electronic engineering

## Abstract

Robust wireless communication using relaying system and Non-Orthogonal Multiple Access (NOMA) will be extensively used for future IoT applications. In this paper, we consider a fall detection IoT application in which elderly patients are equipped with wearable motion sensors. Patient motion data is sent to fog data servers via a NOMA-based relaying system, thereby improving the communication reliability. We analyze the average signal-to-interference-plus-noise (SINR) performance of the NOMA-based relaying system, where the source node transmits two different symbols to the relay and destination node by employing superposition coding over Rayleigh fading channels. In the amplify-and-forward (AF) based relaying, the relay re-transmits the received signal after amplification, whereas, in the decode-and-forward (DF) based relaying, the relay only re-transmits the symbol having lower NOMA power coefficient. We derive closed-form average SINR expressions for AF and DF relaying systems using NOMA. The average SINR expressions for AF and DF relaying systems are derived in terms of computationally efficient functions, namely Tricomi confluent hypergeometric and Meijer’s G functions. Through simulations, it is shown that the average SINR values computed using the derived analytical expressions are in excellent agreement with the simulation-based average SINR results.

## Introduction

Internet of Things (IoT) is the basis of many important future applications for smart healthcare^1^. With IoT, data connectivity among sensors (placed on the patients) and remote servers placed at homes/hospitals for patients’ vital monitoring will be realized. The server will collect large health related data from several patients and run Artificial Intelligence (AI) algorithms to observe anomalies in the vital signs of the patients. For example, an IoT sensor can monitor the blood pressure of a patient and notify in case of an abnormal trend^2^. Other applications can include fall detection of an elderly person using sensors and notify the relatives at home or care providers in the hospital^3^.

Fall detection refers to techniques that help find out the occurrence of a fall event in elderly patients and also notify in case the patient collapses^4^. Fall detection is an important application of smart health care in which patients or elderly persons are equipped with sensors such as motion, doppler or accelerometer^5^. These sensors are placed on a wearble device worn by the patients. The data from these sensors is continuously transmitted to the fog devices placed in the hospitals or homes^6^. The fog nodes collect data from several elderly patients and apply machine learning algorithms to classify data into normal walk or fall event. Moreover, the fog nodes also share their data with a cloud server to develop a global machine learning model^3^.

To enable efficient fall detection, reliable data sharing with high data rates between the IoT sensor devices and fog nodes is critical. This is because the learning algorithm rely on the reliability of shared data. As the reliability of the data is decreased, the accuracy of the learning algorithms can be severely degraded. To overcome this challenge, future wireless communications utilizes techniques such as the use of relay nodes to improve the signal strength at the receiver^7^, and the use of Non-Orthogonal Multiple Access (NOMA)^8^.

Non-orthogonal multiple access (NOMA) is considered as a promising technology that will enable future wireless networks achieve massive connectivity, enhanced spectrum efficiency, energy efficiency, and user-fairness^9^. By utilizing superposition coding and successive interference cancellation (SIC), NOMA can serve multiple users simultaneously with different power levels^10^. This approach results in significant spectral efficiency gains over the conventional orthogonal multiple access (OMA) system^11^. Moreover, unlike the conventional opportunistic user scheduling, NOMA can serve users with different channel conditions reliably^12^.

Cooperative relaying can be beneficial to future IoT networks as it has the ability to provide uninterrupted connectivity to the users whose channel conditions are not favourable. Recently, NOMA-based cooperative relaying system has gained significant research attention due to its ability to further increase the number of supported users and spectral efficiency^13^. In such systems, maximum ratio combining (MRC) is used at the destination to increase the spatial diversity. In addition, cooperative relaying systems can also help in enhancing the spectral efficiency of the network as two data symbols can be obtained at the destination in two time slots. Two common techniques for cooperative relaying include Amplify-and-Forward (AF) and Decode-and-Forward (DF).

In this paper, we derive closed-form expressions of average received Signal to Interference and Noise Ratio (SINR) for NOMA-based AF and DF relayed systems for fall detection application. The average SINR performance is of key importance for the analysis and design of any communication network^14^. The knowledge of the statistics of SINR is useful in determining other important performance metrics such as spectral efficiency, coverage probability and symbol error rates. Moreover, the accurate characterization of the average SINR is essential as it is used to solve various communication network problems, e.g. link budget, user association and power control. The SINR analysis also helps in reliability analysis of communication reliability of different IoT applications such as fall detection.

The major contribution of the paper are summarized below:

- We consider an IoT based fall detection system and propose cooperative communications to improve the connectivity performance between the IoT sensors which act as source and fog devices which are the destination nodes.
- We present an average SINR analysis of the NOMA-based AF relaying system, where the destination implements MRC twice to obtain maximum diversity order. The average SINR expressions are derived for both data symbols which are transmitted in two time slots using the AF relaying mode.
- We also derive average SINR expressions for the data symbols of the NOMA-based DF relaying system, where the destination utilizes a single MRC-based receiver structure to jointly decode the two data symbols.
- Using the derived average SINR expressions for AF and DF relaying systems, we also present the upper bounded ergodic sum rate which is based on the Jensen’s inequality.
- Finally, we present numerical results to validate the analysis carried out in this paper. It has been shown that the results with the analytical expressions match with the results of Monte-Carlo simulations.

## Related works

In this section, we provide an overview of fall detection application, recent work done in the area of IoT based fall detection and recent work done in the area of NOMA based cooperative communications.

### Overview of IoT based fall detection application

**Figure 1:**
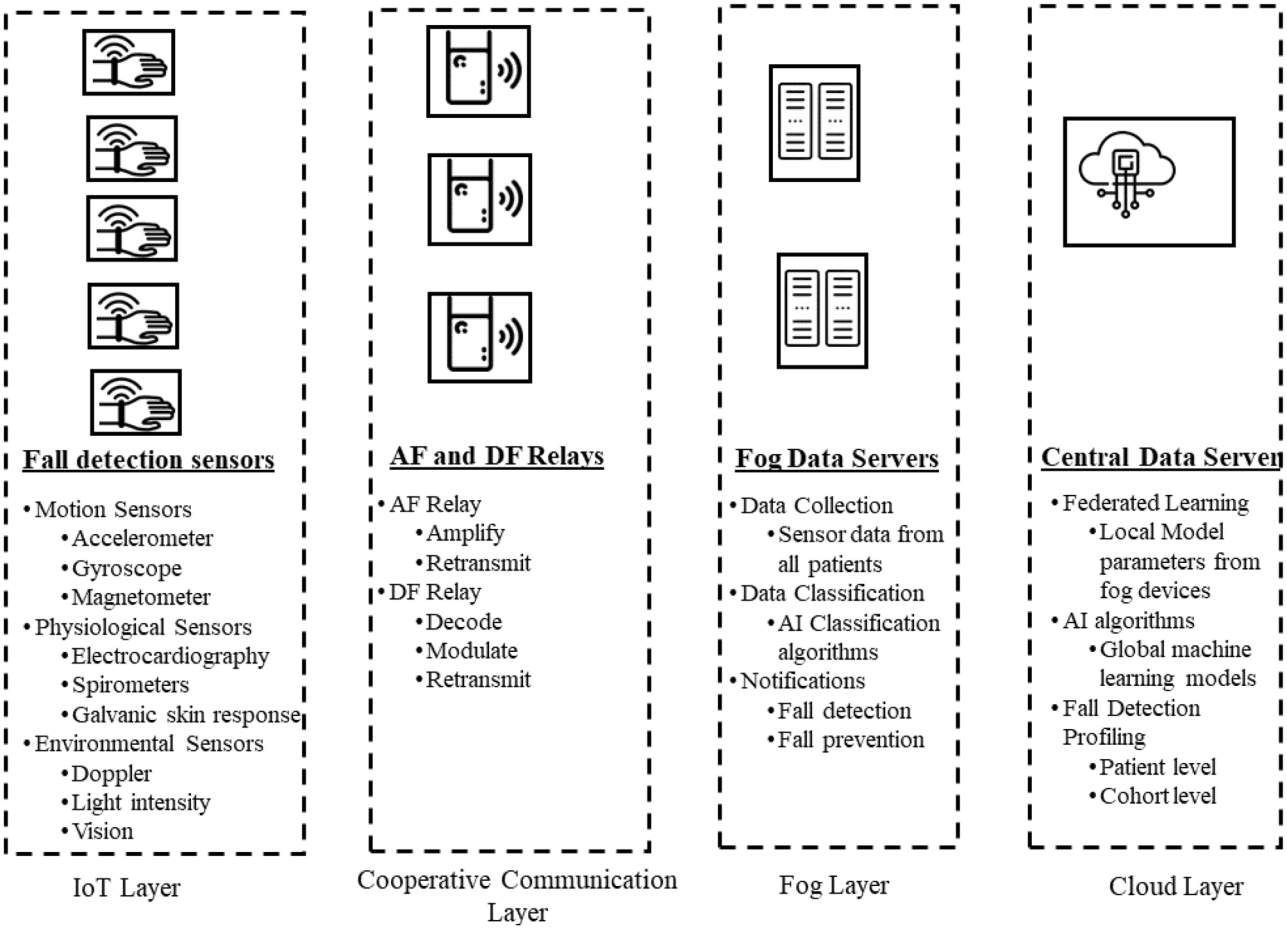
Framework for fall detection application.

The Fall detection application relies on the use of different type of sensors to monitor the movement of patients^5^. In case of an abnormality in the movement, it may further be scrutinized for a fall. In Fig. 1, we present a framework for fall detection application. There are four different layers of the fall detection application describe in the following.

#### IoT layer

The IoT layer involves the wearable device worn by the patients which can have different type of installed sensors. Three kind of sensors are normally used for fall detection. These include the motion sensors, physiological sensors and environmental sensors^3^.

In the motion sensors, the movement of the patient is observed regularly. The sensors in this category can be an accelerometer which measures the velocity of the patient with respect to time. Similarly, a gyroscope can also be used to measure the angular position of the patient. Magnetometers can also track the position of the patients based on magnetic fields. Additionally, GPS sensors and indoor Wi-Fi fingerprinting mechanisms can also be used for fall detection^6^.

The physiological sensors is another category of sensors that can help detection of fall. They rely on change in body parameters of the patient in case of a fall. These include sensors such as electrocardiography to monitor the electrical signals of the heart, spirometers to find the inward and outward flow of air in lungs, and galvanic skin response to measure the conductivity of the skin based on electrical signals^5^.

The wearable device of the patients contain a variety of these sensors and regularly generate patient data which is to be sent to the fog devices installed in the hospitals or houses.

### Cooperative communication layer

The goal of cooperative communication layer is to provide reliability in communications between the IoT devices and the fog devices. This communication can be severly degraded due to multi-path fading of the sender-receiver channel^15^. Hence, small relay nodes can be installed to boost the signal reception at the destination (fog devices). In this regard, AF and DF relays are used. In the AF relay, the sender’s signal is simply amplified and forward towards the destination. On the other hand, in the DF relays, the signal is first decoded, modulated and then retransmitted towards the destination.

#### Fog layer

The Fog layer includes fog nodes placed at several locations in the hospital or homes. The reason of placing these fog servers near the edge is to reduce the transmission latency of the tasks. The purpose of these fog devices is to collect data from different patients, apply machine learning algorithms to detect falls and notify the patients/healthcare providers about the fall. Classification algorithms can be easily applied at this layer which can monitor the regular movements data of the patient and detect abnormalities. Furthermore, complex machine learning algorithms can also be used to develop a local fall detection model.

#### Cloud layer

The data from fog layer can be transmitted towards the central data servers. A federated learning approach may be utilized to share data between the fog layer and the cloud layer. Instead of sharing the raw data with the cloud layer, the fog layer can only share the local fall detection model parameters with the cloud layer. The cloud server upon receiving local fall detection model parameters from many fog devices can apply sophisticated machine learning algorithms to come up with a global fall detection model. The revised model parameters are sent back to the fog devices for update of the local model parameters. The cloud server also conducts fall detection profiling at the patient and cohort level. This corresponds to use of patients’ demographic data such as age, weight etc. and its sensor obtained data to find different correlations for fall detection and prevention.

**Table 1:** Fall detection systems in literature.

References	Sensor/Dataset	Algorithm type	Algorithm execution	Communication technology
^16^	Accelerometer	Threshold based	Cloud	WiFi SMS
^17^	Accelerometer dataset Gyroscope dataset Magentometer dataset	CNN RNN	Device	Bluetooth Cellular
^18^	Accelerometer Doppler Sound	ANN	Fog Cloud	WiFi
^19^	Camera Video	Deep CNN	Cloud	WiFi
^20^	3d Accelerometer	Decision trees ensemble logistic Regression deepnets	Cloud	6 LowPAN
^21^	Accelerometer dataset ECG signal	CNN Clustering	Cloud	WiFi SMS
Proposed framework	Accelerometer	Classifcation	Fog Cloud	Cooperative Communications NOMA based Relaying

### Literature review of IoT based fall detection

Table 1 presents a summary of recent work done related to IoT based fall detection. In^16^, authors utilize accelerometer to measure the position of the patients in real time. Based on the movement data, a threshold based algorithm is developed to detect falls. The threshold algorithm is executed on the cloud server. The communication between the sensors and the cloud is maintained by using WiFi technology and fall detection event notifications are disseminated using SMS.

The work in^17^ utilize a fall detection dataset based on three different sensors namely accelerometer, gyroscope and magnetometer. Based on the datasets, the work utilizes two machine learning algorithms namely Convolution Neural Network (CNN) and Recurrent Neural Network (RNN). Patients are equipped with two werable devices, one at the waits and other at the neck. Both of these algorithms are executed at the wearable device itself and once a fall event is detected, the wearable communicate with each other using Bluetooth. The neck wearable device is a airbag helmet that saves the patient from injury in case of a fall event. Data from the wearble devices is also communicated to the cloud server for further analysis and improve the learning models.

In^18^, authors use three sensors for fall detection, the first is the accelerometer, the second is Doppler sensor and third is sound senors. Data from these sensors is fed to an Artificial Neural Network (ANN) executed on the fog devices and cloud server. Communication is exchanged between the devices using WiFi.

The work in^19^ uses camera to record video of the area in which patients are present. The video data is given as input to a Deep CNN and fall detection events are detected at the cloud. Data from video is shared with the cloud server over WiFi.

In^20^, a 3d accelerometer is used to obtain movement data of the patients. Different machine learning algorithms are used to detect fall events. These algorithms include decision trees, ensemble based techniques, logistic regression and DeepNets. The agorithms are executed at the cloud and sensor to cloud communication is achieved using 6 LowPAN technology.

The work in^21^ uses accelerometer dataset obtained from different online sources and applied CNN and clustering algorithms to detect falls at the cloud. Once the fall occurs, the ECG signal of the patient is observed and notifications are made using SMS.

As compared to the existing frameworks, this paper focuses on a cooperative communication approach for sharing data between sensors and fog nodes. We assume patients are equipped with an accelerometer sensor and this data is needed to be transmitted to the fog nodes so that classificaiton algorithms can be applied.

### Literature review of NOMA based cooperative communications

Many existing studies have investigated the performance gains of cooperative NOMA relay systems in terms of outage probability and ergodic achievable rate. The analysis of the achievable rate for a NOMA based relaying system is performed in^22^ with Rayleigh fading channels and decode-and-forward (DF) relay. The authors in^23^ investigate the ergodic sum rate and outage performance of the NOMA based relaying system. For Rician fading channels, the average achievable rate expression is derived for the NOMA relaying system in^24^ using a DF relay. By employing a relay in amplify-and-forward (AF) mode, authors in^25^ calculate the asymptotic outage probability and ergodic sum rate of the NOMA based relaying system. There are numerous studies^26–31^ that provide the useful performance analysis of NOMA-based communication systems.

NOMA cooperative dual-hop relaying is investigated in^32^, where the selection of the best relay from the set of multiple relays is based on a max-min signal-to-interference-plus-noise ratio (SINR) criteria. Ergodic sum-rate and outage probabilities are derived in^32^ for the DF and AF protocols. The performance of a full duplex relay assisted cognitive radio network with NOMA is evaluated in^33^. The secondary and primary users are coupled using the NOMA transmission strategy, such that a common relay for cooperative transmission. An accurate closed-form expressions for outage probability and average throughput are also presented. It has been shown in^33^ that the full duplex relay assisted cognitive radio network gives superior performance compared to its half duplex counterpart. The impact of imperfect SICs on the performance of space-time block codes (STBC) based cooperative NOMA is investigated in^34^. The closed-form expression of the ergodic capacity with imperfect SICs is derived. The SINR expressions of the two weak-user nodes are derived in^35^ using the AF relay cooperative NOMA model. At high SNR, an asymptotically tight approximation of symbol error rate (SER) is also derived using moment-generating function (MGF). The performance of cooperative NOMA based internet of things (IoT) networks for the generalised non-homogeneous fading channel model is investigated in^36^, where the Meijer’s G-function is used to derive closed-form analytical expressions of outage probability for secondary NOMA users.

## System model

**Figure 2:**
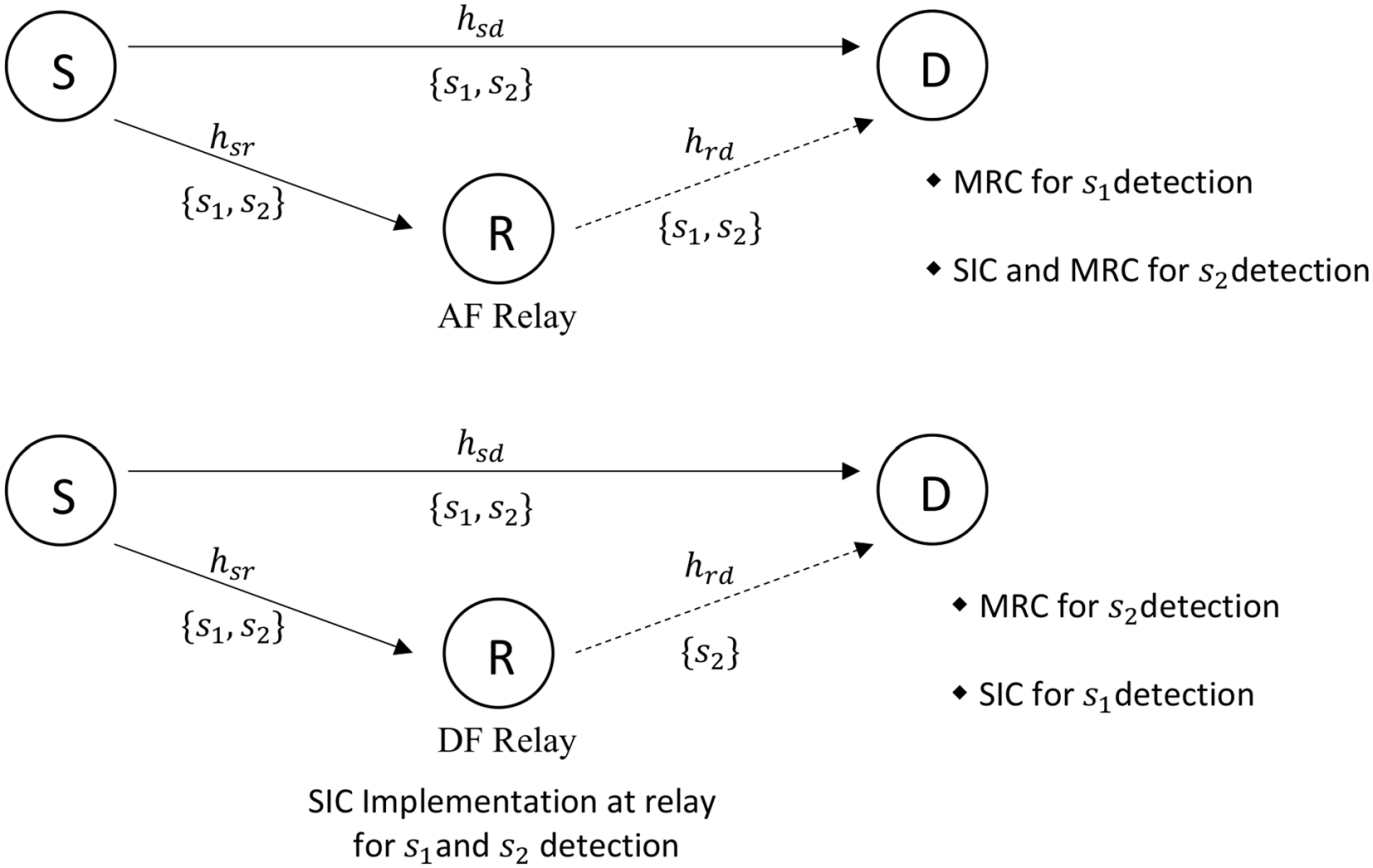
An illustration of the communication for fall detection system model with AF and DF relaying modes (Here S = Motion Sensor Node, R = Relay node, D = fog server Node).

We consider a communication for fall detection application scenario based on Fig. 1. The considered system consists of a motion sensor source, relay as a cooperative communication node, and the fog server destination node, represented by *S*, *R* and *D*, respectively as shown in Fig. 2. For an IoT healthcare application, the source can be the sensor placed on the patient, and the destination can be the data server located in the home or hospital. The relay nodes can be the wireless nodes placed at different places to enhance the data connectivity.

In the considered system, each node consists of a single antenna and it is assumed that the relay operates in a half-duplex mode. The source node transmits two symbols $s_1$ and $s_2$ using the superposition coding $s=\sqrt{Pa_{1}}\,s_{1}+\sqrt{Pa_{2}}\,s_{2}$ at the first time slot, where *P* is the total transmission power at the source. It may be noted that $a_1$ and $a_2$ are NOMA power levels such that $a_1+a_2=1$. It is further assumed that the direct $S \rightarrow D$ link is experiencing severe fading such that the symbol $s_1$ is allocated more power, i.e., $a_1>a_2$^23^. The depiction of the system model is shown in Fig. 2, where the detection of symbols in AF and DF modes are discussed in the following subsections.

### AF relaying

Here, we present the system model of the fall detection application that uses NOMA-based AF relaying system. The system model follows the receiver design of^25^. At the $n^{th}$ time slot, the signal received at the destination and at the relay can be written as

1 $$\begin{aligned} y_{d}^{\text {AF}}[n]=h_{sd}[n]\left( \sqrt{Pa_{1}}\,s_{1}[n]+\sqrt{Pa_{2}}\,s_{2}[n]\right) +w_{d}[n], \end{aligned}$$

and

2 $$\begin{aligned} y_{r}^{\text {AF}}[n]=h_{sr}[n]\left( \sqrt{Pa_{1}}\,s_{1}[n]+\sqrt{Pa_{2}}\,s_{2}[n]\right) +w_{r}[n], \end{aligned}$$

respectively, where $h_{sd}$ and $h_{sr}$ denote the Rayleigh distributed flat fading channels of the source-destination $S \rightarrow D$ and source-relay $S \rightarrow R$ links, respectively. *P* denotes the total transmit power at the source and relay nodes. The additive white Gaussian noise (AWGN) values at the destination and relay for the $n^{\text {th}}$ time slot are denoted by $w_d[n]$ and $w_r[n]$, respectively. For convenience, the noise variances at relay and destination are assumed to be same, represented by $\sigma ^2$.

The relay amplifies the received signal with a gain factor determined as

$A=\sqrt{P/(P|h_{sr}|^2+\sigma ^{2})}$. It may be noted that the destination does not decode the received signal at the $n^{\text {th}}$ time slot rather it waits for the amplified signal transmitted by the relay at the $\left( n+1\right) ^{\text {th}}$ time slot. Therefore, the signal received at the destination during the $\left( n+1\right) ^{\text {th}}$ time slot can be written as

3 $$\begin{aligned} y_{d}^{\text {AF}}[n+1]&=h_{rd}[n+1](Ay_{r}^{\text {AF}}[n])+w_{d}[n+1], \end{aligned}$$

where $h_{rd}$ denotes the Rayleigh distributed flat fading channels of the relay-destination $R \rightarrow D$ link. In order to decode the symbol $s_1$, the signals received at the destination, $y_{d}^{\text {AF}}[n]$ and $y_{d}^{\text {AF}}[n+1]$ are combined using MRC with weighting factors $q_{s_1}[n]$ and $q_{s_1}[n+1]$. Therefore, the combined signal at the destination becomes $y_{d}^{\text {AF}}=q_{s_1}[n]y_{d}^{\text {AF}}[n]+q_{s_1}[n+1]y_{d}^{\text {AF}}[n+1]$. The weighting factors are chosen as

4 $$\begin{aligned} q_{s_1}[n]=\left( \frac{h_{sd}^{*}[n]\sqrt{Pa_{1}}}{|h_{sd}[n]|^{2}Pa_{2}+\sigma ^{2}}\right) , \end{aligned}$$

and

5 $$\begin{aligned} q_{s_1}[n+1]=\left( \frac{h_{rd}^{*}[n+1]Ah_{sr}^{*}[n] \sqrt{Pa_{1}}}{|h_{rd}[n+1]|^{2}A^{2}|h_{sr}[n]|^{2}Pa_{2}+|h_{rd}[n+1]|^{2}A^{2}\sigma ^{2}+\sigma ^{2}}\right) , \end{aligned}$$

where $\left( \cdot \right) ^{*}$ denotes the conjugate transpose. Note that SIC is implemented separately on the received signals of the two time slots in order to remove symbol 1 interference at the destination. To decode the symbol 2, weighting factors are chosen as

6 $$\begin{aligned} q_{s_2}[n]=\left( \frac{h_{sd}^{*}[n]\sqrt{Pa_{2}}}{\sigma ^{2}}\right) , \end{aligned}$$

and

7 $$\begin{aligned} q_{s_2}[n+1]=\left( \frac{h_{rd}^{*}[n+1]Ah_{sr}^{*}[n] \sqrt{Pa_{2}}}{|h_{rd}[n+1]|^{2}A^{2}\sigma ^{2}+\sigma ^{2}}\right) . \end{aligned}$$

From^25^, we can express the combined SINR of the symbol $s_1$ as the sum of the SINR of the direct link, ${\gamma }_{daf-s1}$, and SINR of the relayed link, ${\gamma }_{raf-s1}$, which is given by

8 $$\begin{aligned} {\gamma }_{caf-s1}&= {\gamma }_{daf-s1}+{\gamma }_{raf-s1}\nonumber \\&= \left( \frac{a_1 \gamma _{sd}}{a_2 \gamma _{sd} +1} + \frac{a_1 \gamma _{sr} \gamma _{rd}}{a_2 \gamma _{sr} \gamma _{rd} + \gamma _{sr} + \gamma _{rd} + 1}\right) , \end{aligned}$$

where, $\gamma _{ij}=\frac{P}{\sigma ^{2}}|h_{ij}|^{2}D_{ij}^{-\alpha }$, *i*, *j* $\in$ $\left( s,r,d\right)$ represents the exponentially distributed instantaneous SNR of the corresponding link. $|h_{ij}|^{2}$ is the instantaneous channel gain between the nodes *i* and *j*. $D_{ij}$ is the link distance between the nodes *i* and *j*, and $\alpha$ denotes the path-loss exponent. Similarly, the combined SINR of the symbol $s_2$ can be expressed as the sum of the SINR of the direct link, ${\gamma }_{daf-s2}$, and SINR of the relayed link, ${\gamma }_{raf-s2}$, such that

9 $$\begin{aligned} {\gamma }_{caf-s2}&= {\gamma }_{daf-s2}+{\gamma }_{raf-s2}\nonumber \\&= a_2 \gamma _{sd} + \left( \frac{a_2 \gamma _{sr} \gamma _{rd}}{\gamma _{sr} + \gamma _{rd} + 1}\right) . \end{aligned}$$

The average SINR analysis of both the symbols $s_1$ and $s_2$ for the NOMA-based AF system is presented in Section “SINR analysis of AF relayed system”.

### DF relaying

We utilize the receiver design of^23^ for the NOMA-based DF relaying system in the considered fall detection communications. In the case of the DF relay transmission, the signal received at the fog node destination and relay in the $n^{\text {th}}$ time slot can be written as

10 $$\begin{aligned} y_{d}^{\text {DF}}[n]=h_{sd}[n]\left( \sqrt{Pa_{1}}\,s_{1}[n]+\sqrt{Pa_{2}}\,s_{2}[n]\right) +w_{d}[n], \end{aligned}$$

and

11 $$\begin{aligned} y_{r}^{\text {DF}}[n]=h_{sr}[n]\left( \sqrt{Pa_{1}}\,s_{1}[n]+\sqrt{Pa_{2}}\,s_{2}[n]\right) +w_{r}[n], \end{aligned}$$

respectively. After the first transmission phase, the symbol $s_{1}$ is decoded at the relay which is followed by the detection of the symbol $s_2$ using SIC. During the $(n+1)^{\text {th}}$ time slot, the relay transmits the symbol $s_2$ to the destination. The received signal in the $(n+1)^{\text {th}}$ time slot at the destination is given by

12 $$\begin{aligned} y_{d}^{\text {DF}}[n+1]=h_{rd}[n+1] \sqrt{P}\,s_{2}[n+1] + w_{d}[n+1]. \end{aligned}$$

After the completion of the second transmission phase, the destination employs MRC on the received signals of the two time slots, where the MRC weights are given by

13 $$\begin{aligned} q_{d}[n]=\left( \frac{h_{sd}^{*}[n] P a_{2}}{|h_{sd}[n]|^{2} P a_1 +\sigma ^{2}}\right) , \end{aligned}$$

and

14 $$\begin{aligned} q_{d}[n+1]=\left( \frac{h_{rd}^{*}[n+1] P}{\sigma ^{2}}\right) . \end{aligned}$$

From the resultant signal, the symbol $s_2$ is decoded first at the destination, while treating symbol $s_{1}$ as interference. Using SIC, the symbol $s_1$ is obtained at the destination. Now, we can write the SINR of the symbol $s_{1}$ denoted by $\gamma _{cdf-s2}$, and the SINR of the symbol $s_2$, represented by $\gamma _{cdf-s2}$, at the destination as

15 $$\begin{aligned} {\gamma }_{cdf-s1} = \min \left( \frac{a_1 \gamma _{sr}}{a_2 \gamma _{sr} +1}\,,\,a_1 \gamma _{sd} \right) , \end{aligned}$$

and

16 $$\begin{aligned} {\gamma }_{cdf-s2}=\min \left( a_2 \gamma _{sr}\,,\,\frac{a_2 \gamma _{sd}}{a_1 \gamma _{sd}+1}+ \gamma _{rd} \right) , \end{aligned}$$

respectively. The average SINR analysis of both the symbols $s_1$ and $s_2$ for the NOMA-based DF system is presented in Section “SINR analysis of DF relayed system”.

## SINR analysis of AF relayed system

In this section, we derive the average received SINRs for symbols $s_1$ and $s_2$ in the NOMA-based AF relaying system for fall detection communications. For our analysis, we define $\lambda _{sd}=\frac{P}{\sigma ^2} E\left\{ |h_{sd}|^{2}\right\} D_{sd}^{-\alpha }$, $\lambda _{sr}=\frac{P}{\sigma ^2} E\left\{ |h_{sr}|^{2}\right\} D_{sr}^{-\alpha }$ and $\lambda _{rd}=\frac{P}{\sigma ^2}E\left\{ |h_{rd}|^{2}\right\} D_{rd}^{-\alpha }$ as the average received signal-to-noise ratios (SNRs) for the $S\rightarrow D$, $S\rightarrow R$ and $R\rightarrow D$ links, respectively. We define three random variables denoted by *U*, *V* and *W* representing $\gamma _{sd}$, $\gamma _{sr}$ and $\gamma _{rd}$ respectively. The mean values of the exponentially distributed random variables *U*, *V* and *W* are given by $\lambda _{u}$, $\lambda _{v}$ and $\lambda _{w}$ respectively. For notational convenience, we use $a=a_1$ and $b=a_2$.

### Average SINR for symbol $s_1$

The average received SINR at the destination for the symbol $s_1$ after applying MRC can be determined using the instantaneous SINR (8) as

17 $$\begin{aligned} {\overline{\gamma }}_{caf-s1}&={\overline{\gamma }}_{daf-s1}+{\overline{\gamma }}_{raf-s1}\nonumber \\&=E\left\{ \frac{aU}{bU+1}\right\} +E\left\{ \frac{a VW}{bVW+V+W+1}\right\} \nonumber \\&=E\left\{ X_1\right\} +E\left\{ Y_1\right\} . \end{aligned}$$

It may be noted that ${\overline{\gamma }}_{caf-s1}$, ${\overline{\gamma }}_{daf-s1}$ and ${\overline{\gamma }}_{raf-s1}$ denote the combined, direct and relayed link average SINRs for symbol $s_1$, respectively. The expectations in (17) are solved in the Lemma 1.

#### Lemma 1

Let the random variable $X_1$ be related to *U* through $X_1=\frac{aU}{bU+1}$, then the $E\left\{ X_1\right\}$ can be determined as

18 $$\begin{aligned} E\left\{ X_1\right\} =\frac{a}{b}\,\Psi \left( 1,0,\frac{1}{b\lambda _u}\right) . \end{aligned}$$

Let another random variable $Y_1$ be related to *V* and *W* through $Y_1=\frac{aVW}{bVW+V+W+1}$, then the $E\left\{ Y_1\right\}$ can be calculated as

19 $$\begin{aligned} E\left\{ Y_1\right\}&=a\sum _{n=0}^{\infty }\frac{\left( 1\right) _{n}\left( 2\right) _{n}\left( 2\right) _{n}}{n!}\nonumber \\&\quad \times \left( a\right) ^n\Psi \,\left( 1+n,0,\frac{1}{\lambda _{v}}\right) \,\Psi \,\left( 1+n,0,\frac{1}{\lambda _{w}}\right) , \end{aligned}$$

where $\Psi \left( \cdot ,\cdot ,\cdot \right)$ is the Tricomi confluent hypergeometric function (TCHF)^37, Eq. (9.211.4)^.

#### Proof

Please see Appendix A.

### Average SINR for symbol $s_2$

The average received SINR at the destination for the symbol $s_2$ using (9) can be expressed as

20 $$\begin{aligned} {\overline{\gamma }}_{caf-s2}&={\overline{\gamma }}_{daf-s2}+{\overline{\gamma }}_{raf-s2} \nonumber \\&=E\left\{ bU\right\} +E\left\{ \frac{bVW}{V+W+1}\right\} \nonumber \\&=E\left\{ X_2\right\} +E\left\{ Y_2\right\} , \end{aligned}$$

where ${\overline{\gamma }}_{caf-s2}$, ${\overline{\gamma }}_{daf-s2}$ and ${\overline{\gamma }}_{raf-s2}$ denote the combined, direct and relayed link average SINRs for symbol $s_2$, respectively. The expectations in (20) are solved in the Lemma 2.

#### Lemma 2

Let the random variable $X_2$ *be related to* *U* *through* $X_2=bU$, *then the* $E\left\{ X_2\right\}$ *can be determined as*

21 $$\begin{aligned} E\left\{ X_2\right\} =b\lambda _{u}. \end{aligned}$$

Let another random variable $Y_2$ be related to *V* and *W* through $Y_2$=$\frac{bVW}{V+W+1}$, then the $E\left\{ Y_2\right\}$ can be calculated as22

#### Proof

Please see Appendix B.

## SINR analysis of DF relayed system

In this section, we present the derivation details of the average received SINRs for symbols $s_1$ and $s_2$ in the NOMA relaying system with the DF transmission mode at the relay.

### Average SINR for symbol $s_1$

The average received SINR at the destination for the symbol $s_1$ using (15) can be expressed^23, Eq. (9)^ as

23 $$\begin{aligned} {\overline{\gamma }}_{cdf-s1}&=E\left\{ min\left( \frac{aV}{bV+1}\,,\,aU \right) \right\} \nonumber \\&=E\left\{ Z_1\right\} , \end{aligned}$$

where ${\overline{\gamma }}_{cdf-s1}$ denote the combined link average SINR for $s_1$. The expectation in (23) is worked out in the Lemma 3.

#### Lemma 3

Let the random variable $Z_1$ be related to *U* and *V* through the relation $Z_1=min\left( \frac{aV}{bV+1}\,,\,aU \right)$, then the $E\left\{ Z_1\right\}$ can be calculated as

24 $$\begin{aligned} E\left\{ Z_1\right\}&=\frac{a}{b}\,exp\left( -\frac{1}{b\lambda _{u}}\right) \nonumber \\&\quad \times \sum _{n=0}^{\infty }\frac{\left( \frac{1}{b\lambda _{u}}\right) ^n}{n!}\Psi \left( 1,-n,\frac{1}{b\lambda _{v}}\right) . \end{aligned}$$

#### Proof

Please see Appendix C.

### Average SINR for symbol $s_2$

The average received SINR at destination for symbol $s_2$ using (15) can be expressed^23, Eq. (10)^ as

25 $$\begin{aligned} {\overline{\gamma }}_{cdf-s2}&=E\left\{ min\left( bV\,,\,\frac{bU}{aU+1}+W \right) \right\} \nonumber \\&=E\left\{ Z_2\right\} , \end{aligned}$$

where ${\overline{\gamma }}_{cdf-s2}$ is the combined link average SINR for $s_2$. The expectation in (25) is worked out in the Lemma 4.

#### Lemma 4

Let the random variable $Z_2$ be related to *U*, *V* and *W* through the relation $Z_2=min\left( bV\,,\,\frac{bU}{aU+1}+W \right)$, then $E\left\{ Z_2\right\}$ can be shown to be equal to (26).

26 $$\begin{aligned} E\left\{ Z_2\right\}&= \left( \frac{b}{a}\right) \,exp\left( -\frac{1}{a\lambda _{v}}\right) \Sigma _1 + b\lambda _{v}\left\{ 1-exp\left( -\frac{1}{a\lambda _{v}}\right) \right\} \nonumber \\&\quad +\left\{ \,\frac{b \lambda _{v}\lambda _{w}}{b\lambda _{v} + \lambda _{w}} \exp \,\left( \,-\frac{b \lambda _{v}+\lambda _{w}}{a\lambda _{v} \lambda _{w}}\,\right) \,-\, \left( \frac{b}{a}\right) exp\left( \,\frac{1}{a\lambda _{v}}+\frac{b}{a\lambda _{w}}\,\right) \,\Sigma _2\right\} \Sigma _3 ,\end{aligned}$$

where

$$\begin{aligned} \Sigma _1&= \sum _{k=0}^{\infty }\frac{\left( \frac{1}{a\lambda _{v}}\right) ^k}{k!}\Psi \left( 1,-k,\frac{1}{a\lambda _{u}}\right) \\ \Sigma _2&= \sum _{k=0}^{\infty }\frac{\left( -\frac{1}{a\lambda _{v}}-\frac{b}{a\lambda _{w}}\right) ^k}{k!}\Psi \left( 1,-k,\frac{1}{a\lambda _{u}}\right) \\ \Sigma _3&= \sum _{n=0}^{\infty }\frac{\Gamma \left( 1+n\right) }{n!} \left( \frac{b}{a\lambda _{w}}\right) ^{n}\Psi \left( n,0,\frac{1}{a\lambda _{u}}\right) . \end{aligned}$$

Although not shown here, it has been observed that summations $\Sigma _1$, $\Sigma _2$ and $\Sigma _3$ require few numbers of terms (at least two terms) to converge.

#### Proof

Please see Appendix D.

## Ergodic sum rate analysis

The average received SINRs derived in the previous section can be used to find an upper bound on the sum rate of the system. For this purpose, the Jensen’s inequality is used and we can write the upper bounded sum rate as

27 $$\begin{aligned} {\overline{R}}_{si}&=\frac{1}{2}E\left\{ \log _2\left( 1+\gamma _{cm_j-s_i}\right) \right\} \nonumber \\&\le \frac{1}{2}\log _2\left( 1+E\left\{ \gamma _{cm_j-s_i}\right\} \right) \nonumber \\&=\frac{1}{2}\log _2\left( 1+{\overline{\gamma }}_{cm_j-s_i}\right) , \end{aligned}$$

where $m_j$ represents the mode of the relay i.e., $m_j=\{af,df\}$, such that *af* corresponds to the AF mode and *df* represents the DF mode.

**Figure 3:**
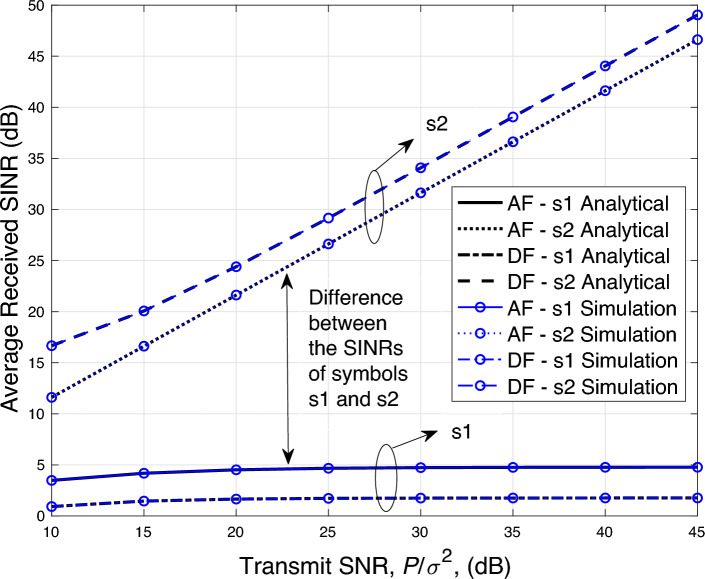
Average SINR versus transmit power performance.

**Figure 4:**
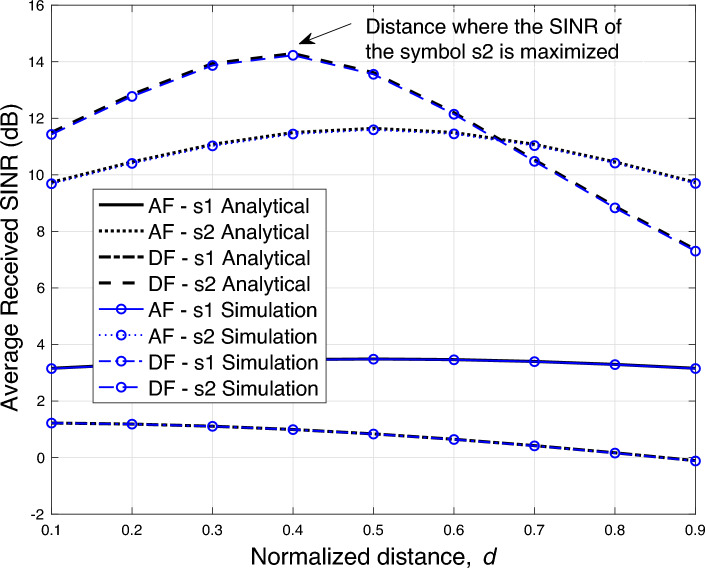
Average SINR versus normalized source-relay distance performance.

**Figure 5:**
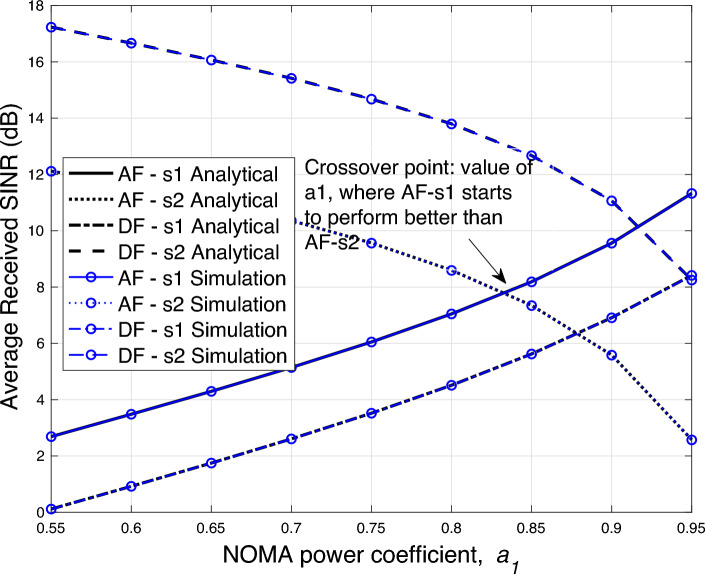
Average SINR versus $a_1$ performance.

**Figure 6:**
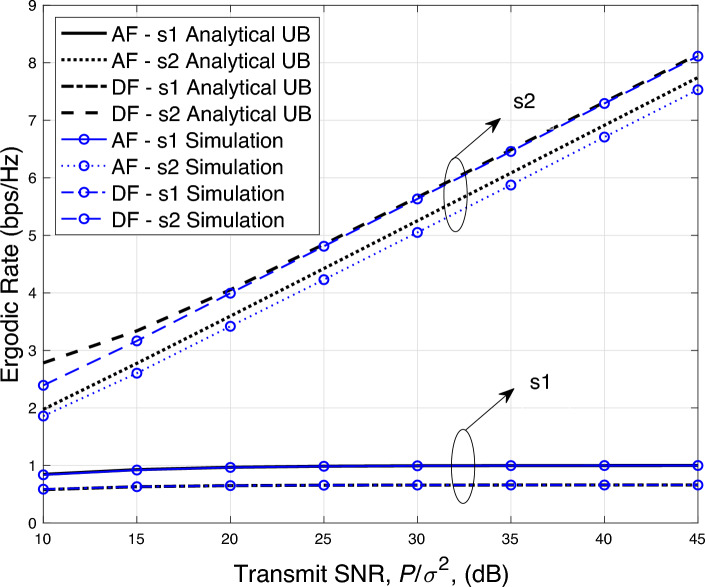
Ergodic rate versus transmit power performance.

## Results and discussion

In this section, we present the average received SINR results for the fall detection communication application using NOMA-based AF and DF relayed systems. The derived analytical results in Sections “SINR analysis of AF relayed system” and “SINR analysis of DF relayed system” are ascertained by the simulation results to verify their correctness. Here, we use $D_{sd}=1$, $D_{sr}=d$ and $D_{rd}=(1-d)$, where $d=\frac{D_{sr}}{D_{sd}}$ is the normalized distance. Furthermore, the path loss exponent is set to $\alpha =3$. The simulation results are obtained by averaging over 1,000,000 independent channel realizations. It is emphasized here that simulation model statistically approximates the analytical model by generating independent channel realizations. The degree of convergence between the analytical and simulation results depends on the number of generated channel realizations. An absolute convergence between the two models can be obtained only when the size of data set of channel realizations approaches to infinity. The transmit SNR $P/\sigma ^2$ is set to 10 dB, unless stated otherwise.

In Fig. 3, we plot the average SINR results (in dB) against the various values transmit SNR as shown in the x-axis. Here, we set $d=0.45$ and $a_1=0.6$, which makes $a_2=0.4$. It can be observed that the derived analytical results fit well with the simulation results, which proves the correctness of the closed form expressions derived in this study for both AF and DF systems. As the transmit SNR is increased, the SINR of the symbol $s_2$ also increases in a linear fashion, while the SINR of $s_1$ only increases marginally. This trend is due to the fact that while decoding $s_1$, the interference from the symbol $s_2$ is treated as a noise. Whereas, SIC is utilized to cancel the interference from $s_1$ while decoding $s_2$.

Fig. 4 shows the average SINR performance by varying the distance between the motion sensor source and relay, i.e., $D_{sr}$. It can be seen that the derived analytical results matches well with the simulation results. We also observe that DF based relaying yields a better SINR performance for the symbol $s_2$ as compared to AF based relaying, when the relay is placed closer to the source. However, as the relay is moved closer to the destination, the SINR with AF based relaying starts to outperform the SINR with DF based relaying for the symbol $s_2$. This is due to the fact that as the relay is placed far away from the source, it receives a further distorted version of the signal and consequently more errors occur during the decoding process. These errors are carried forward to the destination, and therefore, the SINR performance degrades.

In Fig. 5, the average SINR versus $a_1$ performance is shown. As the parameter $a_1$ is increased, the SINR performance gap between the two symbols begins to reduce. The SINR performance with both symbols for the AF case matches when the value of $a_1$ is around 0.83 and after that the SINR performance for the symbol $s_1$ becomes superior. Similarly, for the DF case, the crossover point of the SINR for both the symbols is when $a_1$ reaches 0.95.

Finally, in Fig. 6 we plot the ergodic rate performance against the different values of transmit SNR for both AF and DF based relaying systems. Here, we plot an upper bounded (UB) rate (27) using the derived average SINR results and compares it with the simulation results. It can be seen from Fig. 6 that the UB rates for the symbol $s_1$ for both AF and DF systems are tightly bounded. While the bound is loose for the symbol $s_2$ for both AF and DF based relaying systems.

It is important to note that insights obtained from the SINR results presented in this study are same as the insights acquired from the outage probability results presented in^23,25^ for NOMA-based DF and AF relaying systems, respectively.

### Discussion

From the results, it can be verified that the NOMA-based relay system can enhance the communication reliability for fall detection application. The addition of the cooperative communication layer as proposed in the framework of this paper ensures that the data from the motion sensor is accurately monitored at the fog node destination. This reliable communications is particularly vital in case of an actual fall so that timely response can be taken for the safety of elderly patients. The analytical model presented in this paper can be used to optimize the working of fall detection communication. Important system parameters such as position of relay nodes and transmit power of motion sensor nodes can be optimized such that the reliability of data received by the fog nodes can be maximized and battery life of motion sensors can be improved.

## Conclusions

This study explores the utilization of a relaying system combined with NOMA-based communication for fall detection applications. In this research, we have mathematically derived closed-form expressions to calculate the average SINR for a NOMA-based relaying system employing both AF and DF modes. Through extensive simulations, we have compared these SINR expressions against numerically computed average SINR results, demonstrating a consistent alignment between the derived analytical results and the simulation outcomes. Our findings suggest that the received SINR experiences significant degradation in DF relaying scenarios when the relay’s proximity to the destination is reduced. These performance trends can be effectively tailored to meet specific fall detection system requirements by thoughtfully designing the power levels allocated within the NOMA framework.

## Data Availability

Data is available from the corresponding author upon request.

## Appendix

### Appendix A

We can write $E\left\{ X_1\right\}$ as

28 $$\begin{aligned} E\left\{ X_1\right\} =\frac{a}{b\lambda _{u}}\int _{0}^{\infty }\frac{u}{u+\frac{1}{b}}exp\left( -\frac{u}{\lambda _{u}}\right) \,du, \end{aligned}$$

which can be evaluated using^37, Eq. (3.383.10)^ and^37, Eq. (8.351.4)^, to obtain (18). $\blacksquare$

Similarly, $E\left\{ Y_1\right\}$ can be written as

29 $$\begin{aligned} E\left\{ Y_1\right\} =E\left\{ \frac{aAV}{V+B}\right\} , \end{aligned}$$

where $A=\frac{w}{bw+1}$ and $B=\frac{w+1}{bw+1}$. Now we have

30 $$\begin{aligned} \,E\left\{ Y_1\right\}&\,=\,\frac{a}{\lambda _{v}\lambda _{w}}\int _{0}^{\infty }\,\underbrace{\int _{0}^{\infty }\,\frac{Av}{v+B} \exp \left( \frac{-v}{\lambda _{v}}\right) }_{I_1} exp\left( \frac{-w}{\lambda _{w}}\right) dv dw. \end{aligned}$$

The integral $I_1$ can be evaluated using^37, Eq. (3.383.10)^ and^37, Eq. (8.351.4)^ as

31 $$\begin{aligned} I_1=\lambda _{v}\,\Psi \left( 1,0,\frac{1}{b\lambda _{v}}\times \left( \frac{w}{w+\frac{1}{b}}+\frac{1}{w+\frac{1}{b}}\right) \right) . \end{aligned}$$

After substituting (31) in (30) and putting $x=\frac{w}{b\lambda _{v}w+\lambda _{v}}$, $E\left\{ Y_1\right\}$ becomes

32 $$\begin{aligned} E\left\{ Y_1\right\}&=\frac{a}{b^{2}\lambda _{w}}\int _{0}^{\frac{1}{b\lambda _{v}}}\,\frac{x}{\left( \frac{1}{b\lambda _{v}}-x\right) ^2}\,\Psi \left( 1,0,\frac{1}{\lambda _{v}}+ax\right) \nonumber \\&\quad \times exp\left( -\frac{1}{b\lambda _{w}}\times \frac{x}{\frac{1}{b\lambda _{v}}-x}\right) \,dx, \end{aligned}$$

where, we have used the fact that $1-b=a$ in (32). TCHF in (32) can be expanded by using the identity in [^38^, Eq. (13.13.9)], such that

33 $$\begin{aligned} \Psi \left( 1,0,\frac{1}{\lambda _{v}}+ax\right)&=\frac{1}{a\lambda _{v}}\left( \frac{1}{\frac{1}{a\lambda _{v}}+x}\right) \sum _{n=0}^{\infty }\frac{\left( 1\right) _{n}\left( 2\right) _{n}}{n!}\nonumber \\&\quad \times \Psi \left( 1+n,0,\frac{1}{\lambda _{v}}\right) \left( \frac{x}{\frac{1}{a\lambda _{v}}+x}\right) ^{n}. \end{aligned}$$

Now, substituting (33) in (32), we get

34 $$\begin{aligned} E\left\{ Y_1\right\}&=\frac{1}{b^{2}\lambda _{v}\lambda _{w}}\sum _{n=0}^{\infty }\frac{\left( 1\right) _{n}\left( 2\right) _{n}}{n!}\,\Psi \left( 1+n,0,\frac{1}{\lambda _{v}}\right) \nonumber \\&\quad \times \int _{0}^{\frac{1}{b\lambda _{v}}} \,\frac{x^{n}}{\left( \frac{1}{a\lambda _{v}}+x\right) ^{n+1}}\frac{x}{\left( \frac{1}{b\lambda _{v}}-x\right) ^2}\nonumber \\&\quad \times exp\left( -\frac{1}{b\lambda _{w}}\times \frac{x}{\frac{1}{b\lambda _{v}}-x}\right) \,dx. \end{aligned}$$

Substituting $t= \frac{x}{\frac{1}{b\lambda _{v}}-x}$ in (34) and re-arranging, as

35 $$\begin{aligned} E\left\{ Y_1\right\}&=\frac{a}{b\lambda _{w}}\sum _{n=0}^{\infty }\frac{\left( 1\right) _{n}\left( 2\right) _{n}}{n!}\left( a\right) ^n\Psi \left( 1+n,0,\frac{1}{\lambda _{v}}\right) \nonumber \\&\quad \times \underbrace{ \int _{0}^{\infty } \,\left( \frac{t}{b+t}\right) ^{n+1}exp\left( -\frac{1}{b\lambda _{w}}t\right) \,dt}_{I_2}. \end{aligned}$$

Note that the functions inside $I_2$ can be expressed in terms of Fox’s H-function [?, Eq. (1.1.1)] as

36

where $\left( \frac{\frac{t}{b}}{1+\frac{t}{b}}\right) ^{n+1}=H^{1,1}_{1,1}\left[ \frac{t}{b}|\cdot \right]$ is obtained using [?, Eq. (2.9.5)] and [?, Eq. (2.1.5)] whereas $exp\left( -\frac{1}{b\lambda _{w}}t\right) =H^{1,0}_{0,1}\left[ \frac{t}{b\lambda _{w}}|\cdot \right]$ is obtained from [?, Eq. (2.9.4)]. The integral $I_2$ can be solved using [?, Eq. (2.8.4)] to give

37 $$\begin{aligned} I_{2}&= \frac{b\lambda _{w}}{\Gamma \left( 1+n\right) }H^{1,2}_{2,1}\left[ \lambda _{w}\bigg |\begin{array}{c} \left( 1,1\right) ,\left( 0,1\right) \\ \left( n+1,1\right) \end{array}\right] \nonumber \\&=\frac{b\lambda _{w}}{\Gamma \left( 1+n\right) }G^{1,2}_{2,1}\left( \lambda _{w}\bigg |\begin{array}{c} 1,0 \\ n+1 \end{array}\right) , \end{aligned}$$

wherein the Fox’s H-function reduces to the Meijer’s G-function using the relation [?, Eq. (2.9.1)].. Meijer’s G-function can be transformed into the TCHF to yield $I_2$ as

38 $$\begin{aligned} I_{2}=b\lambda _{w}\,\Gamma \left( 2+n\right) \,\Psi \left( 1+n,0,\frac{1}{\lambda _{w}}\right) . \end{aligned}$$

We obtain the final expression for $E\left\{ Y_1\right\}$ using the identity^37, Eq. (8.339.1)^ and substituting $I_2$ in (35), yielding (19) which completes the proof. $\blacksquare$

39 $$\begin{aligned} {\overline{F}}_{Z_{2}}\left( x\right) = {\left\{ \begin{array}{ll} exp\left( - \frac{x}{b \lambda _{v}} - \frac{x}{\lambda _{w}} \right) \underbrace{\frac{1}{\lambda _{u}}\int _{0}^{\infty }exp\left( \frac{ub}{\left( ua+1\right) \lambda _{w}}\right) \,exp\left( -\frac{u}{\lambda _{u}}\right) \,du}_{I_6}, &{} \ \ \text {if } x > \frac{b}{a}\\ exp\left( - \frac{x}{b \lambda _{v}} - \frac{x}{\left( b - a x\right) \lambda _{u}} + exp\left( - \frac{x}{b \lambda _{v}} \right) - exp\left( - \frac{x}{b \lambda _{v}} - \frac{x}{\lambda _{w}} - \frac{x}{\left( b -a x\right) \lambda _{u}} \right) \right) I_6, &{} \ \ \text {if } x < \frac{b}{a} \end{array}\right. } \end{aligned}$$

### Appendix B

The expectation $E\left\{ Y_2\right\}$ can be computed as

40 $$\begin{aligned} E\left\{ Y_2\right\} =&\frac{b}{\lambda _{v}\lambda _{w}}\int _{0}^{\infty }\,\underbrace{\int _{0}^{\infty }\,\frac{v}{v+w+1} \,exp\left( -\frac{v}{\lambda _{v}}\right) \,dv}_{I_3} \nonumber \\&\times w\,exp\left( -\frac{w}{\lambda _{w}}\right) \,dw. \end{aligned}$$

The integral $I_3$ can be evaluated using^37, Eq. (3.383.10)^ and^37, Eq. (8.351.4)^.

Substituting $I_3$ in (40), we get

$$\begin{aligned} E\left\{ Y_2\right\} =&\frac{b}{\lambda _{w}}\int _{0}^{\infty }\,w\,\Psi \,\left( 1,0,\frac{w}{\lambda _{v}}\,+\,\frac{1}{\lambda _{v}}\right) exp\left( -\frac{w}{\lambda _{w}}\right) \,\,dw . \end{aligned}$$

Now using the integral representation of TCHF^37, Eq. (9.211.4)^ w.r.t *t*, $E\left\{ Y_2\right\}$ can be re-written as

41 $$\begin{aligned} E\left\{ Y_2\right\} =&\frac{b}{\lambda _{w}}\int _{0}^{\infty }\,\left( 1+t\right) ^{-2}exp\left( -\frac{t}{\lambda _{v}}\right) \nonumber \\&\times \underbrace{\int _{0}^{\infty }w \,exp\left( -\frac{t w}{\lambda _{v}}-\frac{w}{\lambda _{w}}\right) \,dw}_{I_4}\,dt. \end{aligned}$$

The integral $I_4$ is evaluated using^37, Eq. (3.326.2)^, giving

42 $$\begin{aligned} I_4=\lambda _{v}^2\,\left( t+\frac{\lambda _{v}}{\lambda _{w}}\right) ^{-2}. \end{aligned}$$

Substituting $I_4$ in (41) and re-arranging terms, we get

$$\begin{aligned} E\left\{ Y_2\right\}&=b\lambda _{w}\int _{0}^{\infty }\,exp\left( -\frac{t}{\lambda _{v}}\right) \left( 1+t\right) ^{-2}\left( 1+t \frac{\lambda _{w}}{\lambda _{v}}\right) ^{-2}dt. \end{aligned}$$

The $E\left\{ Y_2\right\}$ can be further expressed in terms of Fox’s H-function $\left( 1+t\right) ^{-2}$=$H^{1,1}_{1,1}\left[ t|\cdot \right]$ and $\left( 1+t\frac{\lambda _{w}}{\lambda _{v}}\right) ^{-2}$=$H^{1,1}_{1,1}\left[ t\frac{\lambda _{w}}{\lambda _{v}}|\cdot \right]$ by using the identity [?, Eq. (2.9.1)].

43 $$\begin{aligned} E\left\{ Y_2\right\}&=b\lambda _{w}\,\int _{0}^{\infty }\,exp\left( \frac{-t}{\lambda _{v}}\right) H^{1,1}_{1,1}\,\left[ t\bigg |\,\begin{array}{c} \left( -1,1\right) \\ \left( 0,1\right) \end{array}\,\right] H^{1,1}_{1,1}\,\left[ t\frac{\lambda _{w}}{\lambda _{v}}\bigg |\,\begin{array}{c} \left( -1,1\right) \\ \left( 0,1\right) \end{array}\,\right] dt. \end{aligned}$$

The above integral can be evaluated using [?, Eq. (2.6.2)] in terms of Fox’s H-function of two variables which can be reduced to Meijer’s G-function of two variables by using [?, Eq. (2.3.1)]. This will yield (22) which completes the proof. $\blacksquare$

### Appendix C

The complementary cumulative distribution function (CCDF) ${\overline{F}}_{Z_{1}}$ of $Z_{1}$ is given by

44 $$\begin{aligned} {\overline{F}}_{Z_{1}}\left( x\right)&=Pr\left[ aU \,>\,x,\,\frac{aV}{bV+1}\,>\,x\right] \nonumber \\&=Pr\left[ aU>x\right] \, Pr\left[ \frac{aV}{bV+1}>x\right] , \end{aligned}$$

where we have exploited the statistical independence of *U* and *V* in the second expression. Since ${\overline{F}}_{Z_{1}}\left( x\right) =0$ for $x\ge \frac{a}{b}$, therefore, the CCDF within the range $0<x<\frac{a}{b}$ can be written as^23, Eq. (12)^

45 $$\begin{aligned} {\overline{F}}_{Z_{1}}\left( x\right) =exp\left( -\frac{x}{a\lambda _{u}}-\frac{x}{\lambda _{v}\left( a-bx\right) }\right) . \end{aligned}$$

Now $E\left\{ Z_1\right\}$ can be expressed as

46 $$\begin{aligned} E\left\{ Z_1\right\}&=\int _{0}^{\frac{a}{b}}exp\left( -\frac{x}{a\lambda _{u}}-\frac{x}{\lambda _{v}\left( a-bx\right) }\right) \, dx, \end{aligned}$$

where we have used the fact that ${\overline{F}}_{Z_{1}}\left( x\right) =1-F_{Z_{1}}\left( x\right)$, wherein $F_{Z_{1}}\left( x\right)$ is the cumulative distribution function (CDF) and $E\left\{ Z_{1}\right\}$=$\int _{0}^{\infty }\left( 1-F_{Z_{1}}\left( x\right) \right) dx$. Now (46) can be simplified by substituting $a-bx=at$ and using the series expansion of $exp\left( \frac{t}{b\lambda _{u}}\right)$ to get

47 $$\begin{aligned} E\left\{ Z_1\right\}&=\frac{a}{b}\,exp\left( \frac{1}{b\lambda _{v}}\,-\,\frac{1}{b\lambda _{u}}\right) \,\sum _{n=0}^{\infty }\frac{\left( \frac{1}{b\lambda _{u}}\right) ^n}{n!} \underbrace{\,\int _{0}^{1}\,t^{n}exp\left( \frac{-1}{b\lambda _{v}\,t}\right) \,dt}_{I_5}. \end{aligned}$$

We simplify the integral $I_5$ by making the following change of variable $\frac{1}{b\lambda _{v}\,t}=u$, such that

48 $$\begin{aligned} I_5=\left( \frac{1}{b\lambda _{v}}\right) ^{n+1}\int _{\frac{1}{b\lambda _{v}}}^{\infty }u^{-n-2}exp\left( -u\right) du. \end{aligned}$$

The integral $I_5$ can be evaluated using^37, Eq. (3.381.6)^ and employing the identity in [?, Eq. (13.14.5),, we get

49 $$\begin{aligned} I_5=\,exp\left( -\frac{1}{b\lambda _{v}}\right) \,\Psi \left( 1,-n,\frac{1}{b\lambda _{v}}\right) . \end{aligned}$$

Substituting $I_5$ in (47), we get (24) and the proof is complete. $\blacksquare$

### Appendix D

The CCDF ${\overline{F}}_{Z_{2}}$ of $Z_{2}$ can be written as (39) (at the top of the page)^23, Eq. (14)^. First, we evaluate the integral $I_6$ as

50 $$\begin{aligned} I_{6}=\frac{1}{\lambda _{u}}\,\int _{0}^{\infty }exp\left( \frac{ub}{\left( ua+1\right) \lambda _{w}}\right) \,exp\left( -\frac{u}{\lambda _{u}}\right) \,du. \end{aligned}$$

A simplified expression of (50) can be obtained by expanding $exp\left( \frac{ub}{\left( ua+1\right) \lambda _{w}}\right)$ in series as

51 $$\begin{aligned} I_{6}\,=\,\frac{1}{\lambda _{u}}\sum _{n=0}^{\infty }\,\frac{\left( \frac{b}{a\lambda _{w}}\right) ^{n}}{n!} \,\int _{0}^{\infty }\,\left( \frac{ua}{ua+1}\right) ^{n}\,exp\left( -\frac{u}{\lambda _{u}}\right) \,\,du. \end{aligned}$$

Similar to the solution of $I_2$, the above integral can be evaluated by following the steps through (36)–(38), to get

52 $$\begin{aligned} I_{6}=\sum _{n=0}^{\infty }\frac{\Gamma \left( 1+n\right) }{n!} \left( \frac{b}{a\lambda _{w}}\right) ^{n}\Psi \left( n,0,\frac{1}{a\lambda _{u}}\right) . \end{aligned}$$

The rest of the expressions in (39) can be divided in four parts as $E\left\{ Z_{2-1}\right\}$, $E\left\{ Z_{2-2}\right\}$, $E\left\{ Z_{2-3}\right\}$ and $E\left\{ Z_{2-4}\right\}$. The first part can be expressed as

$$\begin{aligned} E\left\{ Z_{2-1}\right\} =\int _{0}^{\frac{b}{a}}exp\left( -\frac{x}{b\lambda _{v}}-\frac{x}{\lambda _{u}\left( b-ax\right) }\right) \,dx \end{aligned}$$

We benefit from the calculations through (46)–(49) to solve $E\left\{ Z_{2-1}\right\}$ as

53 $$\begin{aligned} E\left\{ Z_{2-1}\right\}&=\frac{b}{a}\,exp\left( \frac{-1}{a\lambda _{v}}\right) \sum _{k=0}^{\infty }\frac{\left( \frac{1}{a\lambda _{v}}\right) ^k}{k!}\Psi \left( 1,-k,\frac{1}{a\lambda _{u}}\right) . \end{aligned}$$

Similarly, for the second part, we have

54 $$\begin{aligned} E\left\{ Z_{2-2}\right\}&\,=\,\int _{0}^{\frac{b}{a}}\,exp\left( \frac{-x}{b\lambda _{v}}\right) \,dx =b\lambda _{v}\left\{ 1-exp\left( \frac{-1}{b\lambda _{v}}\right) \right\} . \end{aligned}$$

The third part is given by

$$\begin{aligned} E\left\{ Z_{2-3}\right\} \,=\,\int _{0}^{\frac{b}{a}}\,exp\left( -\left\{ \frac{1}{b\lambda _{v}}+\frac{1}{\lambda _{w}}\right\} x\right) exp\left( -\frac{x}{\lambda _{u}\left( b-ax\right) }\right) \,dx \end{aligned}$$

Again we use (46)-(49) to solve $E\left\{ Z_{2-3}\right\}$ as

55 $$\begin{aligned} E\left\{ Z_{2-3}\right\}&=\left( \frac{b}{a}\right) \,exp\left( \frac{1}{a\lambda _{v}}+\frac{b}{a\lambda _{w}}\right) \nonumber \\&\times \sum _{k=0}^{\infty }\frac{\left( -\frac{1}{a\lambda _{v}}-\frac{b}{a\lambda _{w}}\right) ^k}{k!}\Psi \left( 1,-k,\frac{1}{a_1\lambda _{u}}\right) . \end{aligned}$$

Finally, the fourth part is evaluated as

56 $$\begin{aligned} E\left\{ Z_{2-4}\right\}&= \int _{\frac{b}{a}}^{\infty } exp\left( - \frac{x}{b \lambda _{v}} - \frac{x}{\lambda _{w}} \right) \nonumber \\&=\frac{b \lambda _{v}\lambda _{w}}{b\lambda _{v} + \lambda _{w}} \exp \left( -\frac{b \lambda _{v}+\lambda _{w}}{a\lambda _{v} \lambda _{w}}\right) . \end{aligned}$$

After evaluating $I_6$, $E\left\{ Z_{2-1}\right\}$, $E\left\{ Z_{2-2}\right\}$, $E\left\{ Z_{2-3}\right\}$ and $E\left\{ Z_{2-4}\right\}$, we get $E\left\{ Z_2\right\}$ (26) and proof is completed.  $\blacksquare$

## References

[1] Malik UM, Javed MA, Zeadally S, Islam SU (2022). Energy-efficient fog computing for 6g-enabled massive IoT: Recent trends and future opportunities. IEEE Internet of Things J..

[2] Ahmed J, Nguyen TN, Ali B, Javed A, Mirza J (2022). On the physical layer security of federated learning based IoMT networks. IEEE J. Biomed. Health Inform..

[3] Karar ME, Shehata HI, Reyad O (2022). A survey of IoT-based fall detection for aiding elderly care: Sensors, methods, challenges and future trends. Appl. Sci..

[4] Zhang Y, Zheng X, Liang W, Zhang S, Yuan X (2022). Visual surveillance for human fall detection in healthcare IoT. IEEE Multimed..

[5] Mozaffari N, Rezazadeh J, Farahbakhsh R, Yazdani S, Sandrasegaran K (2019). Practical fall detection based on IoT technologies: A survey. Internet of Things.

[6] Wang X, Ellul J, Azzopardi G (2020). Elderly fall detection systems: A literature survey. Front. Robot. AI.

[7] Vu T-H, Nguyen T-T, Pham Q-V, da Costa DB, Kim S (2023). A novel partial decode-and-amplify NOMA-inspired relaying protocol for uplink short-packet communications. IEEE Wireless Commun. Lett..

[8] Zhang N, Zhu X (2023). A hybrid grant NOMA random access for massive MTC service. IEEE Internet of Things J..

[9] Ding Z (2017). Application of non-orthogonal multiple access in LTE and 5G networks. IEEE Commun. Mag..

[10] Al-Obiedollah H, Salameh HAB, Cumanan K, Ding Z, Dobre OA (2023). Self-sustainable multi-IRS-aided wireless powered hybrid TDMA-NOMA system. IEEE Access.

[11] Khan WU, Ihsan A, Nguyen TN, Ali Z, Javed MA (2022). Noma-enabled backscatter communications for green transportation in automotive-industry 5.0. IEEE Trans. Ind. Inform..

[12] Islam SMR, Avazov N, Dobre OA, Kwak K (2017). Power-domain non-orthogonal multiple access (NOMA) in 5G systems: Potentials and challenges. IEEE Commun. Surv. Tutor..

[13] Zeng M, Hao W, Dobre OA, Ding Z (2020). Cooperative NOMA: State of the art, key techniques, and open challenges. IEEE Netw..

[14] Simon MK, Alouini M-S (2005). Digital Communications Over Fading Channels.

[15] Salem A, Musavian L, Jorswieck EA, Aïssa S (2020). Secrecy outage probability of energy-harvesting cooperative NOMA transmissions with relay selection. IEEE Trans. Green Commun.Network..

[16] Nooruddin S, Milon Islam M, Sharna FA (2020). An IoT based device-type invariant fall detection system. Internet of Things.

[17] Mohammad Z, Anwary AR, Mridha MF, Shovon MSH, Vassallo M (2023). An enhanced ensemble deep neural network approach for elderly fall detection system based on wearable sensors. Sensors.

[18] Ribeiro O, Gomes L, Vale Z (2022). IoT-based human fall detection system. Electronics.

[19] Vaiyapuri T (2021). Internet of things and deep learning enabled elderly fall detection model for smart homecare. IEEE Access.

[20] Yacchirema D, de Puga JS, Palau C, Esteve M (2019). Fall detection system for elderly people using IoT and ensemble machine learning algorithm. Pers. Ubiquitous Comput..

[21] Al Dujaili MJ, Dhaam HZ, Mezeel MT (2023). An intelligent fall detection algorithm for elderly monitoring in the internet of things platform. Multimed. Tools Appl..

[22] Kim J-B, Lee I-H (2015). Capacity analysis of cooperative relaying systems using non-orthogonal multiple access. IEEE Commun. Lett..

[23] Xu M, Ji F, Wen M, Duan W (2016). Novel receiver design for the cooperative relaying system with non-orthogonal multiple access. IEEE Commun. Lett..

[24] Jiao R, Dai L, Zhang J, MacKenzie R, Hao M (2017). On the performance of NOMA-based cooperative relaying systems over Rician fading channels. IEEE Trans. Veh. Technol..

[25] Abbasi O, Ebrahimi A, Mokari N (2019). Noma inspired cooperative relaying system using an AF relay. IEEE Wireless Commun. Lett..

[26] Do D-T (2020). UAV relaying enabled NOMA network with hybrid duplexing and multiple antennas. IEEE Access.

[27] Do D-T, Nguyen T-L, Rabie KM, Li X, Lee BM (2020). Throughput analysis of multipair two-way replaying networks with NOMA and imperfect CSI. IEEE Access.

[28] Do D-T, Nguyen M-SV, Le A-T, Rabie KM, Zhang J (2020). Joint full-duplex and roadside unit selection for NOMA-enabled v2x communications: Ergodic rate performance. IEEE Access.

[29] Tian X (2019). Performance analysis of two-way relay NOMA systems with hardware impairments and channel estimation errors. KSII Trans. Internet Inf. Syst..

[30] Rabie KM, Adebisi B, Yousif EHG, Gacanin H, Tonello AM (2017). A comparison between orthogonal and non-orthogonal multiple access in cooperative relaying power line communication systems. IEEE Access.

[31] Rabie, K. *et al.* On the performance of non-orthogonal multiple access over composite fading channels. arXiv:2004.07860 (2020).

[32] Huang R (2020). Performance analysis of NOMA-based cooperative networks with relay selection. China Commun..

[33] Babu, V. S., Deepan, N. & Rebekka, B. Performance analysis of cooperative full duplex NOMA system in cognitive radio networks. In *Proc. International Conference on Wireless Communications Signal Processing and Networking (WiSPNET)*, 84–87. 10.1109/WiSPNET48689.2020.9198341 (2020).

[34] Akhtar, M. W., Hassan, S. A. & Jung, H. Ergodic capacity analysis of Alamouti coded cooperative communication in downlink NOMA. In *Proc. IEEE Global Communications Conference*, 1–6. 10.1109/GLOBECOM42002.2020.9322511 (2020).

[35] Zhu, D. *et al.* Analysis of asymptotically tight approximation SER for cooperative NOMA systems. In *IEEE 19th International Conference on Communication Technology (ICCT)*, 34–39. 10.1109/ICCT46805.2019.8947023 (2019).

[36] Arzykulov S (2020). Hardware- and interference-limited cognitive IoT relaying NOMA networks with imperfect SIC over generalized non-homogeneous fading channels. IEEE Access.

[37] Gradshteyn IS, Ryzhik IM (2007). Table of Integrals, Series and Products.

[38] Olver FW, Lozier DW, Boisvert RS, Clark CW (2010). NIST Handbook of Mathematical Functions.

